# Flow FL-100 Transcranial Direct Current Stimulation (tDCS) Used at Home for People with Self-Reported Depression: Impact on Self-Reported Concentration Problems

**DOI:** 10.1192/bjo.2026.11054

**Published:** 2026-06-30

**Authors:** Chris Griffiths, Aria Banazadeh, Mu Mu

**Affiliations:** 1 Northamptonshire Healthcare NHS Foundation Trust, Northampton, United Kingdom; 2 University of Northampton, Northampton, United Kingdom

## Abstract

**Aims:**

There is a high prevalence of concentration and cognitive functioning problems in people with depression. Research evidence indicates that transcranial direct current stimulation (tDCS) can improve cognitive functioning, and an aspect of cognitive functioning is the ability to concentrate. Flow FL-100 is a tDCS device self-administered at home.

The aim was to investigate the impact of 1, 2, 3, 6 and 10 weeks of Flow Neuroscience AB FL-100 tDCS use on self-reported concentration problems in people with depression, using the Montgomery–Åsberg Depression Rating Scale Self (MADRS-S) concentration problem question.

**Methods:**

A retrospective analysis of Montgomery–Åsberg Depression Rating Scale (MADRS-S) self-report data collected between 2020 and 2024.

**Results:**

Out of 20,197 tDCS users with self-reported depression at baseline, 10,888 had concentration problems (53.9%). Among those who adhered to the tDCS protocol, theproportion who moved from having concentration problems to not having them was 29.3% at one week, 37% at two weeks, 45.2% at three weeks, 54.7% at six weeks, and 57.4% at ten weeks. Concentration problems strongly correlate with depression.

**Conclusion:**

The results show that tDCS can reduce concentration problems in those with self-reported depression. Some people experience concentration problems despite addressing lifestyle and environmental factors that can negatively affect concentration. There are negative side effects of some methods of improving concentration, for example, the use of stimulants. tDCS could be a valuable alternative approach to reducing concentration problems in people experiencing depression. Concentration problems are symptoms of other mental illness diagnosis, for example, schizophrenia and tDCS could be used. Appropriately designed and powered randomised controlled trials (RCTs) are warranted to investigate the impact of tDCS on cognitive functioning and concentration.

